# *Asparagopsis armata* Exudate Cocktail: The Quest for the Mechanisms of Toxic Action of an Invasive Seaweed on Marine Invertebrates

**DOI:** 10.3390/biology10030223

**Published:** 2021-03-14

**Authors:** Carla O. Silva, Tiago Simões, Rafael Félix, Amadeu M.V.M. Soares, Carlos Barata, Sara C. Novais, Marco F.L. Lemos

**Affiliations:** 1 MARE—Marine and Environmental Sciences Centre, ESTM, Instituto Politécnico de Leiria, 2520-641 Peniche, Portugal; carla.o.silva@ipleiria.pt (C.O.S.); tiago.simoes@ipleiria.pt (T.S.); rafael.felix@ipleiria.pt (R.F.); sara.novais@ipleiria.pt (S.C.N.); 2 Department of Biology and CESAM (Centre for Environmental and Marine Studies), University of Aveiro, 3810-193 Aveiro, Portugal; asoares@ua.pt; 3 Environmental Chemistry Department, Institute of Environmental Assessment and Water Research (IDAEA) Consejo Superior de Investigaciones Científicas (CSIC), Jordi Girona 18-26, 08034 Barcelona, Spain; cbmqam@cid.csic.es

**Keywords:** biomarkers, fatty acid profile, halogenated compounds, oxidative stress, red macroalgae, secondary metabolites

## Abstract

**Simple Summary:**

The invasive red seaweed *Asparagopsis armata* exhibits a strong invasive behavior, producing harmful secondary metabolites that negatively affect the surrounding community. This study addressed the antioxidant defenses, oxidative damage, and a neuronal parameter, as well as the fatty acid composition responses to sublethal concentrations of *A. armata* released compounds on the marine snail *Gibbula umbilicalis* and the shrimp *Palaemon serratus*. Results revealed that the test species had different metabolic responses to the *A. armata* exudate concentrations tested. Impacts in *G. umbilicalis* does not seem to arise from oxidative stress or neurotoxicity, while for *P. elegans*, an inhibition of AChE and the decrease of antioxidant capacity and increase of LPO suggest neurotoxicity and oxidative stress as contributing to the observed toxicity. Additionally, there were different fatty acid profile changes between species, but omega-3 PUFAs ARA and DPA increased in both invertebrates, indicating a common regulation mechanism of inflammation and immunity responses.

**Abstract:**

The seaweed *Asparagopsis armata* exhibits a strong invasive behavior, producing halogenated compounds with effective biological effects. This study addresses the biochemical responses to sublethal concentrations of *A. armata* exudate on the marine snail *Gibbula umbilicalis* whole body and the shrimp *Palaemon elegans* eyes and hepatopancreas. Antioxidant defenses superoxide dismutase (SOD) and glutathione-S-transferase (GST), oxidative damage endpoints lipid peroxidation (LPO) and DNA damage, the neuronal parameter acetylcholinesterase (AChE), and the fatty acid profile were evaluated. Results revealed different metabolic responses in both species. Despite previous studies indicating that the exudate affected *G. umbilicalis’* survival and behavior, this does not seem to result from oxidative stress or neurotoxicity. For *P. elegans*, the inhibition of AChE and the decrease of antioxidant capacity is concomitant with the increase of LPO, suggesting neurotoxicity and oxidative stress as contributor mechanisms of toxicity for this species. Fatty acid profile changes were more pronounced for *P. elegans* with a general increase in polyunsaturated fatty acids (PUFAs) with the exudate exposure, which commonly means a defense mechanism protecting from membrane disruption. Nonetheless, the omega-3 PUFAs arachidonic acid (ARA) and docosapentaenoic acid (DPA) increased in both invertebrates, indicating a common regulation mechanism of inflammation and immunity responses.

## 1. Introduction

More than 3800 halogenated compounds are known to exist [1], and many are known to be present in the environment, having both biogenic and anthropogenic sources. The largest source of biogenic organohalogens are seaweeds, sponges, corals, tunicates, and bacteria [1]. Some seaweeds produce an array of organohalogens, which exhibit important and vital ecological roles as defense compounds [2].

*Asparagopsis armata*, a species of the family Bonnemaisoniaceae is a red seaweed native to western Australia, and currently is distributed throughout Europe in the Atlantic and Mediterranean basin. This seaweed is known to form specialized cells, known as vesicle or gland cells, which are sources of these halogenated products, including halomethanes, haloalkanes, haloacids, and haloketones [3,4], reported to have potent biological effects to protect themselves from attacks by herbivores and pathogens [5,6], which may ultimately influence diversity of habitats where this seaweed is present [7].

The eutrophication and the occurrence of algal blooms may result in negative ecological consequences to the aquatic ecosystem. Algal blooms of some seaweeds, such as *A. armata*, which are present in shallow waters, can invade several benthic environments and be present or stranded in rock pools and release high concentrations of halogenated compounds, which can be harmful and compromise inhabiting biota [7,8]. Limited information is available on effects of macroalgae exudates’ secondary metabolites in aquatic environments. However, some negative effects of these compounds on aquatic ecosystems can be found in the literature, e.g., [8]. Besides suppressing the growth of other algae [7,9], macroalgae exudates can affect the development and grazing of invertebrates [6] and even vertebrates [10].

In this study, *Gibbula umbilicalis* (da Costa, 1778) and *Palaemon elegans* (Rathke, 1837), abundant species on upper intertidal zone of rocky shores where *A. armata* often occurs and which have wide geographical distributions [11], were used as testing model species to assess the impacts of *A. armata* on coastal communities through a biomarker mechanistic approach.

Once these invertebrates are exposed to contaminants such as *A. armata* exudates, these compounds are likely to go through biotransformation reactions, stimulating the production of reactive oxygen species (ROS) which can damage cellular macromolecules [12] in the form of lipid peroxidation (LPO) and DNA strand breaks. Key antioxidant enzymes that protect cells against ROS include superoxide dismutase (SOD), representing the primary defense against oxygen toxicity, which is responsible for the transformation of O^2−^ into H_2_O_2_ [13]. Glutathione-S-transferase (GST) plays a role in the second phase of the detoxification process, where it facilitates the excretion of xenobiotics [14]. Environmental stressors may also promote neurotoxicity. Acetylcholinesterase, involved in the synaptic transmission of nerve impulse through the hydrolysis of neurotransmitter acetylcholine into choline and acetate, is known to be inhibited by contaminants such as pesticides [15,16,17].

Fatty acid profile (FAP) has also been used as a biochemical response to pollutant exposure [18,19,20,21]. Fatty acids (FAs) are amongst the main constituents of the cell membrane and are involved in a wide range of biological pathways, from the production and permeability of cell membrane to lipids main components, while also being signaling mediators and used as fuel in all metabolic systems, having an important role on neural levels of biochemical and physiological response and in the processes of detoxification and inflammation [18]. This biomarker approach allows for a mode-of-action assessment of *Asparagopsis armata* exudates impact in organisms and eventual repercussions in higher levels of biological organization, such as population or even community levels [22].

The present study aims to address potential mechanisms of action involved in the previously observed toxic effects on two rock-pool invertebrate species, the marine snail *Gibbula umbilicalis* and the shrimp *Palaemon elegans*, exposed to the exudate of *A. armata* [8], consisting mostly of bromoform and dibromoacetic acid [5]. This was performed by assessing oxidative damage (lipid peroxidation and DNA damage), antioxidant and detoxification enzymes (superoxide dismutase and glutathione S-transferase), neuronal activity (acetylcholinesterase), and fatty acid profile changes.

## 2. Materials and Methods

### 2.1. Test Organisms

The collection of *Palaemon elegans* and *Gibbula umbilicalis* was done in the spring in an intertidal rocky shore (Carreiro de Joannes), in Peniche, central Portugal (39°21′18.0″ N, 9°23′40.6″ W), where at the time, *A. armata* was not present. Organisms were acclimated for 7d in natural, filtered seawater, and the temperature was kept at 20 ± 1 °C, with a photoperiod of 16-h:8-h (light:dark), and constant aeration, to maintain conditions as close to natural as possible. Tanks were cleaned daily and every two days the organisms were fed *ad libitum* with small fragments of mussels for the shrimps and *Ulva lactuca* for snails.

### 2.2. Experimental Setup

*Asparagopsis armata* collection, preparation of exudates, and experimental design followed previous work by Silva et al. [8]. Briefly, after collection from Berlenga Island (Peniche) by SCUBA, the seaweeds were gently washed with seawater to remove epibionts, sand and debris. Four aquaria, each containing 50 L of filtered seawater (0.45 μm pore cellulose acetate membrane filters) and 5 kg of *A. armata* were placed for 12 h in the dark at 20 °C. Then, the seaweed was removed, and the water pooled and filtered through 0.45 μm pore cellulose acetate membrane filters and kept at −20 °C until further use (exudate) in PET bottles. As in Silva et al. [8], the produced exudate constitutes the stock solution for all experiments, and experimental concentrations are presented as % of the exudate produced.

After the acclimation period (7 days), the organisms were randomly transferred to glass sampling flasks, and the dilutions of exudate used were: 0, 0.04, 0.07, 0.14, 0.25, 0.47, and 0.87% for the sea snails and 0, 0.11, 0.21, 0.39, 0.72, 1.33, and 2.46% for shrimp (highest concentration for both ranges based on half the LC10 [7] and control treatment (0%) with just seawater). Exposure media was obtained by adding corresponding exudate percentages to natural filtered (0.45 μm) seawater (*v*/*v*). Exposures lasted for 168 h, and 16 *G. umbilicalis*, per treatment, were placed individually in 120 mL glass flasks, and 8 *P. elegans*, per treatment, were placed individually in 600 mL glass flasks. Solutions were renewed daily to avoid excreta accumulation and possible loss of volatile compounds. At the end of the exposure period, organisms were dissected and kept at −80 °C until further analysis.

### 2.3. Biochemical Analysis

#### 2.3.1. Tissue Preparation

To perform the biomarker analysis, the whole soft tissue was used for *G. umbilicalis* [17,19,23], while for *P. elegans*, since this organism is easier to dissect for specific organs with reported activities, the hepatopancreas was used for oxidative stress and fatty acid endpoints [24], and the eyes were used for neuromuscular endpoints [25].

Pools of two snails’ whole-body soft tissue (each pool considered as one biological replicate, *n* = 8) were homogenized in a proportion of 1:12 (m:v) of potassium phosphate buffer (0.1 M, pH 7.4). The homogenate was then divided into three microtubes and kept at −80 °C until further analysis of DNA damage, fatty acid profile (FAP), and lipid peroxidation (LPO). For the latter, sampling microtubes contained BHT (2.6-dieter-butyl-4-metylphenol) 4% in methanol to prevent tissue oxidation. The rest of the homogenate was separated into two microtubes that followed different centrifugations: (1) centrifuged for 5 min at 3000× *g* (4 °C) for the analysis of AChE activity on the resulting supernatant; and (2) centrifuged for 20 min at 10,000× *g* (4 °C) to obtain the postmitochondrial supernatant (PMS), for posterior analysis of SOD and GST activities in a more purified fraction and with less interferents for the antioxidant biochemical assays.

For the *P. elegans* samples (*n* = 8), eyes were homogenized in 300 µL of potassium phosphate buffer (0.1 M, pH 7.4) and centrifuged for 5 min at 3000× *g* (4 °C) for the analysis of AChE activity on the resulting supernatant. Shrimp hepatopancreas was also homogenized in potassium phosphate buffer in a proportion 1:10 (w:v) and divided into different microtubes for the analysis of LPO, DNA damage, and FAP. The remaining homogenate was centrifuged for 20 min at 10,000× *g* (4 °C) to obtain the PMS for posterior analysis of SOD and GST activities.

Resulting microtubes were kept at −80 °C until the respective analysis. In every assay, reaction blanks were performed using K-phosphate buffer instead of the sample and all biomarker spectrophotometric measurements were performed in triplicates at 25 °C using a Synergy H1 hybrid multimode microplate reader (BioTek Instruments, Winooski, VT, USA).

#### 2.3.2. Protein Quantification

The protein concentration, needed for normalization of measured parameters, was quantified in the respective resulting supernatant following Bradford [26], adapted for 96-well flat bottom plate [23]. Briefly, 290 µL of Biorad Bradford solution was added to each sample replicate (10 µL) and incubated for a 15 min period (light protected and at room temperature). After this, the absorbance was measured at 600 nm. Bovine γ-globulin (BGG, 1 mg mL^−1^, Sigma-Aldrich, St. Louis, MO, USA) was used as protein standard, and the results were expressed in mg of protein mL^−1^.

#### 2.3.3. Antioxidant and Detoxification Defenses

GST activity was determined by the method of Habig et al. [27] adapted to microplate [28]. Briefly, the assay was started with the addition of 250 µL of a final reaction mixture containing 200 mM phosphate buffer (pH 6.5), 60 mM CDNB, and 10 mM reduced glutathione, to 50 µL of PMS sample, and then the conjugation of GSH with 1-chloro-2,4-dinitrobenzene (CDNB) was followed at 340 nm for 3 min. GST activity was calculated, using a molar extinction coefficient of 9.6 × 10^−3^ M^−1^ cm^−1^, and expressed in nmol min^−1^ mg^−1^ of protein.

SOD activity was measured according to McCord and Fridovich [29], adapted to microplate, using the xanthine/xanthine oxidase mediated reduction of cytochrome C. Briefly, 100 µL of 50 mM phosphate buffer (pH 7.8) was added to 50 µL of PMS sample in each microplate well, followed by the addition of 50 µL of 0.14 mM xanthine, 50 µL of 60 mM cytochrome C, and 50 µL of 10 mU mL^−1^ xanthine oxidase. The decrease of the cytochrome C reduction was followed at 550 nm for 10 min, and SOD activity was expressed in U mg^−1^ of protein using a SOD standard of 1.5 U mL^−1^, where 1 U represents the amount of enzyme required to inhibit the rate of reduction of cytochrome C by 50%.

#### 2.3.4. Oxidative Damage

The LPO levels were determined by measuring the content of thiobarbituric acid reactive substances (TBARS) by following the methods of Ohkawa et al. [30] and Bird and Draper [31] with the adaptations of Torres et al. [32]. In short, samples were deproteinized with 12% trichloroacetic acid followed by the addition of 0.73% 2-thiobarbituric acid (TBA). After heating the mixture at 100 °C for 1 h, samples were centrifuged at 11,500 *g* for 5 min, and LPO levels were measured in the supernatant at 535 nm. Results were expressed in nmol TBARS g^−1^ ww (wet weight), using a molar extinction coefficient of 1.56 × 10^5^ M^−1^ cm^−1^.

DNA damage (strand breaks) analysis was based on the DNA alkaline precipitation assay [33] adapted from de Lafontaine et al. [34]. In brief, tissue homogenates (50 μL) were incubated with 500 μL of 2% SDS solution containing 50 mM NaOH, 10 mM Tris, 10 mM EDTA, and 500 μL of 0.12 M KCl at 60 °C for 10 min. After placing samples on ice for 15 min to induce the precipitation of SDS associated nucleoproteins and genomic DNA, they were centrifuged at 8000 *g* (4 °C) for 5 min to enhance precipitation. The DNA kept in the supernatant was then linked with Hoescht dye (1 µg mL^−1^ bis-benzimide, Sigma-Aldrich, St. Louis, MO, USA), allowing the estimation of damage levels by fluorescence, using an excitation/emission wavelength of 360/460 nm. Results were expressed as μg g^−1^ ww of DNA using calf thymus DNA as standard to extrapolate DNA concentration.

#### 2.3.5. Neuromuscular Biomarker

The AChE activity was determined according to the methodology proposed by Ellman et al. [35] adapted to microplate for these species, as described in more detail in [17,25]. The absorbance was read at 414 nm for 5 min, monitoring the formation of 5-thio-2-nitrobenzoate anion (TNB), which results from the reaction of 5,5′-dithiobis-(2-nitrobenzoic acid) (DTNB) with thiocholine, a product of the acetylcholine substrate hydrolysis performed by AChE. Results are expressed in nmol min^−1^mg^−1^ of protein using a molar extinction coefficient of 13.6 × 10^3^ M^−1^ cm^−1^.

#### 2.3.6. Fatty Acid Profile

The methodologies for FA preparation and analysis were performed according to Silva et al. [19], with minor modifications. Briefly, for the initial saponification step, 150 µL of 2 M KOH (diluted in 67% ethanol; *v*/*v*) was added to 150 µL of homogenate. Samples were then kept at 80 °C for 1 h, cooled to room temperature, diluted 1:1 with water, acidified (HCl), and FAs isolated with hexane.

To the FA fractions isolated from each sample after saponification, 1.5 mL of acetyl chloride:methanol (1:20 *v*/*v*) solution was added for the derivatization step, and samples were kept at 80 °C for 1 h. After adding, 1 mL of Mili-Q water and 1 mL of hexane for phase separation, the organic layer was recovered to clean GC vials and solvent was evaporated in a vacuum concentrator (Speedvac™) for 10 min. Samples were then resuspended in 50 µL of hexane, and methylated nonadecanoic acid (50 µL; 10 mg mL^−1^) was added as an internal standard to each sample, prior to gas chromatography analysis. Fatty acid methyl ester mixes (PUFA No1 from marine source and PUFA No 3 from Menhaden oil) were used as external standards (Supelco, Bellefonte, PA, USA). Operating conditions were as described by Silva et al. [19]. Theoretical correction factor (FCT) for FID detectors was applied in FA quantification, according to Guo [36].

### 2.4. Statistical Analysis

All statistical analyses were run using R software (version 3.6.3) [37] in combination with user interface RStudio 1.2.5033 [38]. Boxplot graphs were prepared in Graphpad Prism version 7 for Mac (GraphPad Software, San Diego, CA, USA). Both biomarker and fatty acid data were checked for normality with Shapiro–Wilk test (“shapiro.test” function), with most variables having *p* < 0.05 (indicative of non-normal distribution). Thus, Kruskal–Wallis one-way analysis of variance test was performed (“kruskal.test” function) to determine significant differences between exudate treatments for biomarkers and for every fatty acid detected (*p* < 0.05). For significantly different variables, this was followed by a post hoc Nemenyi Test (“kwAllPairsNemenyiTest” function), package PMCMRplus [39], to find which specific treatments significantly differed from control. For the metabolic shift analysis, mean values of each FA that presented significant differences upon exposure to *A. armata* exudate was normalized to the respective mean of the control, and a Bray–Curtis dissimilarity matrix was computed (“vegdist” function), package vegan [40], that was used in the production of the cluster analysis (“agnes” function, package cluster [41]) that can be seen aside the heatmaps (the clustering method used was unweighted pair group method with arithmetic mean, UPGMA, and function to produce heatmap was “heatmap” from base package stats). To visualize the different levels of expression, a degree of color was assigned where green represents the lowest amount of fatty acid, passing through black and ending in red, as a higher amount of fatty acid.

## 3. Results

### 3.1. Biochemical Biomarkers

**Figure 1:**
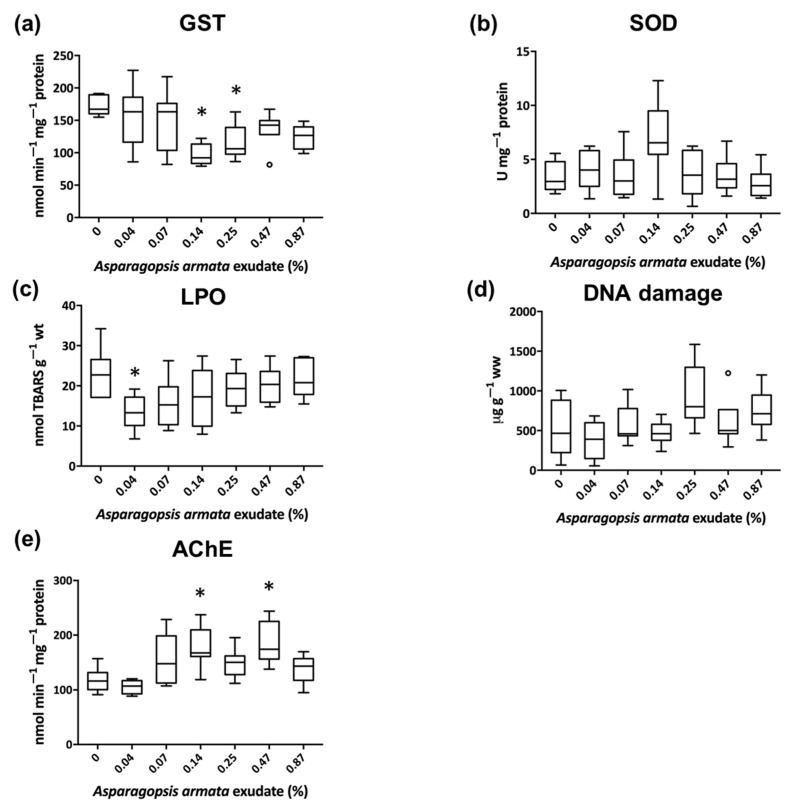
Results of detoxification and antioxidant defenses (**a**,**b**), oxidative damage (**c**,**d**), and acetylcholinesterase (**e**) biomarkers in *Gibbula umbilicalis’* whole body when exposed to *Asparagopsis armata* exudate for 168 h (*n* = 8, pools of 2 individuals). Results are shown in boxplot (i.e., the median, the first and the third quartiles, and the nonoutliers’ range and the outliers); * Significant differences from the control (Kruskal–Wallis test, Nemenyi post hoc test, *p* < 0.05).

**Figure 2:**
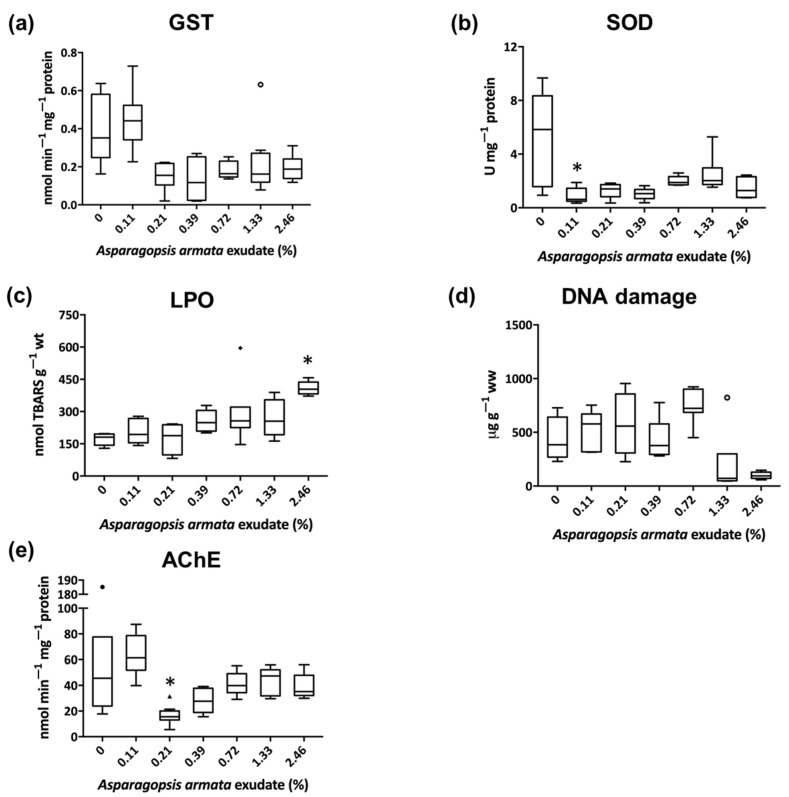
Results of detoxification and antioxidant defenses (**a**,**b**), oxidative damage (**c**,**d**), in *Palaemon elegans*’ hepatopancreas tissue and acetylcholinesterase (**e**) in the eyes, when exposed to *Aparagopsis armata* exudate for 168 h (*n* = 8). Results are shown in boxplot (i.e., the median, the first and the third quartiles, and the nonoutliers’ range and the outliers); * Significant differences from the control (Kruskal–Wallis, Nemenyi, *p* < 0.05).

Measurements of antioxidant defenses, oxidative damage, and neuromuscular biomarkers in gastropod whole body (*G. umbilicalis*) and shrimp tissues (*P. elegans*) are illustrated in Figure 1 and Figure 2, respectively. In general, *A. armata* exudate induced biochemical alterations in both *G. umbilicalis* and *P. elegans* metabolism, yet with different trends of effects between species.

The antioxidant and detoxification enzymes evaluated in the PMS fraction revealed a significant decrease in GST activity in *G. umbilicalis* at 0.14% (C3; *p* = 0.001) and 0.25% (C4; *p* = 0.015) concentrations of *A. armata* exudate, but for SOD, no significant differences were found (Figure 1a,b). Concerning the parameters addressing oxidative damage, a significant decrease was observed in peroxidation of lipids at lower exudate concentration (0.04%), (C1; *p* = 0.041) but with no effects for DNA-strand breaks being registered (Figure 1c,d). Neuromuscular parameter (AChE) had significant higher activities at 0.14 (C3; *p* = 0.021) and 0.47% (C5; *p* = 0.025) of *A. armata* exudate (Figure 1e).

The biochemical analysis of the shrimp *P. elegans*, revealed no significant differences detected for GST, but a decrease in SOD measured in the PMS was observed at 0.11% (C1; *p* = 0.028) of *A. armata* exudate concentration (Figure 2a,b). Regarding the oxidative damage, significant effects of the exudate were observed in LPO at the highest exudate concentration of exposure (C6; *p* = 0.002) and with a trend to increase through consecutive concentrations (Figure 2c). As observed for *G. umbilicalis*, no damage was observed in DNA strands for *P. elegans* (Figure 2d). Additionally, an inhibition of the neuromuscular parameter AChE was identified at 0.21% of *A. armata* exudate (C2; *p* = 0.032) (Figure 2e).

### 3.2. Fatty Acid Profile

**Figure 3:**
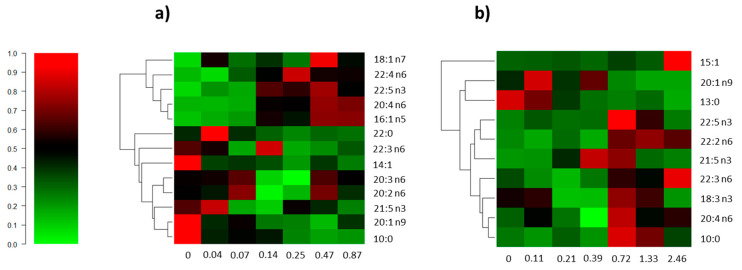
Heatmap depicts an overall comparison of the significantly different fatty acids in *Gibbula umbilicalis* (**a**) and *Palaemon elegans* (**b**) based on their sensitivity to different concentrations of *Asparagopsis armata* exudate. Rows are clustered using Bray–Curtis distances and unweighted pair group method with arithmetic mean (UPGMA) clustering method, and color scaling (relative units) was performed by row after normalization of each fatty acid to the mean of the respective control. On the XX axis are the concentrations of *A. armata* exudate in percentage (%).

Globally, the tissues of the two invertebrates presented FAs ranging from capric acid (10:0) to nervonic acid (24:1 n9), in a total of 43 different FAs detected and identified throughout this study. The complete list of fatty acids found for the different treatments and for both species tested can be consulted in Tables S1 and S2 (Supplementary data). After exposure to *A. armata* exudate, both invertebrates presented significant alterations in concentrations of some FAs, compared to respective control treatments (Tables S1 and S2; Supplementary data). Fatty acids from both invertebrates presenting significant differences in at least one of the exudate treatments (17 FA in total) were selected to be further discussed and were presented in Figure 3. To streamline the overall interpretation, these FAs were clustered according to similarities in their concentration levels across the different exudate treatments. Additionally, these FAs were grouped into their respective FA types for each species, and differences between exposure and control treatments were evaluated for each class (Tables S3 and S4; Supplementary data).

Heatmaps depicts an overall comparison of 13 significantly different fatty acids for *G. umbilicalis* (Figure 3a) and 10 for *P. elegans* (Figure 3b), indicative that these were more responsive to the exudate. It should be noted that there is a generalized shift in lipid metabolism between concentration 0.07% (C2) and 0.14% (C3) for *G. umbilicalis* and between concentrations 0.39% (C3) and 0.72% (C4) for *P. elegans*. When considering all the FAs that presented significant differences to control, more unsaturated FAs tend to increase, and more saturated FAs tend to decrease for both species along treatments, with differences observed at increasing concentrations of the exudate. For instance, a significant reduction in SFAs was denoted for *G. umbilicalis* at 0.25% and 0.87% exudate, while PUFAs increased significantly at 0.47% exudate (Table S3). A similar pattern was denoted for *P. elegans*, where SFAs decreased significantly in four of the six tested concentrations compared to control. The opposite trend was observed for PUFAs, with n6 FAs increasing significantly at 0.72% and 2.46% exudate (Table S4). In *G. umbilicalis*, clustering analysis separated FAs into two major groups. Vaccenic (18:1 n7; *p* = 0.045), adrenic (AdA; 22:4 n6; *p* = 0.01), docosapentaenoic (DPA; 22:5 n3; *p* = 0.00), arachidonic (ARA; 20:4 n6; *p* = 0.00), and hexadecenoic (16:1 n5; *p* = 0.00) acids comprise the first cluster. When compared to control, there is an increase in metabolic levels of these FAs, generally starting from 0.14% (C3) of exudate. The opposite was observed for the second cluster, composed by behenic acid (22:0, *p* = 0.020), 22:3 n6 (*p* = 0.007), myristoleic acid (14:1; *p* = 0.024), dihomo-gamma-linolenic acid (DGLA; 20:3 n6; *p* = 0.018), eicosadienoic acid (EDA; 20:2 n6; *p* = 0.037), heneicosapentaenoic acid (HPA; 21:5 n3; *p* = 0.032), eicosenoic acid (EA; 20:1 n9; *p* = 0.031), and decanoic acid (10:0; *p* = 0.008). Here, there is a general decrease in metabolic levels of these FAs and especially after the 0.14% concentration (C3). Exceptionally, n6 DGLA and eicosadienoic acids presented an increase in C5 (0.47%; *p* = 0.018 and *p* = 0.037, respectively), and 22:3 n6 also increased in C3 (0.14%; *p* = 0.008).

For *P. elegans*, three main clusters were identified, in which the first one is composed by pentadecenoic acid (15:1), remaining invariable throughout treatments but significantly higher for the higher concentration (C6; *p* = 0.002). In the second cluster, EA (20:1 n9; *p* = 0.001) and tridecanoic acid (13:0; *p* = 0.000) presented higher concentrations in control and first treatments, with further depletion in higher exudate concentrated treatments. However, the third cluster, which comprises mostly PUFAs: DPA (22:5 n3; *p* = 0.004), docosadienoic acid (22:2 n6; *p* = 0.008), HPA (21:5 n3; *p* = 0.003), 22:3 n6 (*p* = 0.004), alpha-linoleic acid (ALA; 18:3 n3; *p* = 0.022), ARA (20:4 n6; *p* = 0.034), and decanoic acid (10:0; *p* = 0.006), demonstrated an inverse behavior to the previous cluster, through overall increased FA quantities with increasing concentrations of the exudate (from 0.72%, C4). The concentrations of over half of the FAs in this cluster decreased at C6.

## 4. Discussion

Red algae are a rich source of halogens, predominantly bromine and iodine [8]. In the case of *A. armata*, the major natural products known are numerous halogenated metabolites which possess a wide range of volatility and solubility [3]. The overall toxicity of halogen-containing compounds seems to be derived from their abilities as alkylating agents [3] or by inducing ROS production [42]. Moreover, the alkylating agents, such as haloacetones found in *A. armata*, are well-known enzyme inhibitors, capable of crosslinking serine and histidine residues in various proteins [3].

Here, the activity of the enzyme GST on the marine snail was significantly decreased in mid exudate concentrations. An inhibition of GST is often found as result of an increased level of produced ROS, which among other damage, also inhibits enzymes, and has previously been reported for other marine snails, e.g., [43]. In the present case, this inhibition may also be due to the aforementioned direct action of the exudate compounds on the enzyme [3], while some allelochemicals of plants are also known to act as GST inhibitors [44]. Owing their toxicity to a myriad of secondary metabolites, including more than 100 halogenated compounds [3], exudate dilutions represent a cocktail of different compounds present at different concentrations. These different treatments may trigger differentiated mechanisms of action at the same time, thus resulting in irregular dose-responses [8] or even nonmonotonic dose responses, as discussed in [45,46] becoming factors that increase the need for a less straightforward analysis of organism responses along concentrations.

Regarding oxidative damage parameters for *G. umbilicalis*, the non-observed effects in SOD are concomitant with the non-observed induced damage in both DNA and lipids. LPO levels actually decreased with the snail exposure to lower concentrations of the exudate. The rationale for this decrease, also seen in a vast number of other studies, e.g., [47,48], is yet not clear and entails further research and careful analysis.

The neuronal parameter AChE is involved in the regulation of the transmission of nerve impulses, and contaminants such as chlorpyrifos have been demonstrated to inhibit AChE activity in *G. umbilicalis* [17]. However, in this work, a significant induction of AChE was observed in *G. umbilicalis* exposed to 0.14 and 0.47% exudate concentrations. Reddy et al. [49] also found an increase in the enzyme activity on crab *Barytelphusa guerini* after 4 d of exposure to fluoride, a halogenated compound. The induction of AChE activity after macroalgae exudate exposure may, as discussed by Badiou et al. [50], result from an increased release of hippocampal ACh which may induce a regulatory overcompensation by increasing AChE in the organism cholinergic system [51]. A further explanation is the release of membrane AChE which may trigger a de novo synthesis of the enzyme to replenish the AChE removed from the surface of the cellular membrane [52].

Regarding the shrimp *P. elegans*, an overproduction of ROS [53] and/or exudate compounds’ enzyme inhibitory action [3] might have been responsible for the reported inhibition of SOD activity and the trend for a decreased level of GST, although not significant. This inhibition often leads to the accumulation of ROS, which in turn leads to an increase of LPO [54]. These results are also in agreement with the study of Box et al. [55], where the invasive red macroalgae *Lophocladia lallemandii* induced the increase of MDA levels generated by lipid peroxidation to the bivalve *Pinna nobilis*. Notwithstanding, and although alkylating agents are present in some *A. armata* extracts and have been proven to possess genotoxic properties [3], DNA damage was not found in the present study.

In *P. elegans*, AChE was inhibited at 0.21% of exudate exposure. In the literature, methanolic extracts from *Sargassum* sp. and *Gracilaria gracilis* showed a tendency to inhibit the fish Nile tilapia cholinesterases [56]. Moreover, Custódio et al. [57] have shown that extracts of *A. armata* had potent inhibitory capacity on AChE (58.4% at 10 mg mL^−1^) of human cells. Typically, this enzyme inhibition is known as an early sign of behavioral impairments. Although no effects were seen for feeding activity after the same exudate exposure for 96h [8], this enzyme inhibition may disclose potential higher-level behavioral effects in longer exposures, not addressed in the present study.

The different biomarker responses found for the two species may be linked to the different range of sublethal concentrations used, since they were made considering the survival effects—half the LC_10_ as higher concentration [8]. Nevertheless, one cannot disregard that different tissues were used in the addressed biomarkers. In the shrimp, hepatopancreas was used for oxidative stress and eyes for AChE, while the whole soft tissue was used for the snail, with the latter being a less targeted approach, which may consequently lead to a more diluted response. Altogether, this may explain the more pronounced oxidative and neurotoxic effects seen in *P. elegans*.

Many studies have shown that pollution can change the composition of FAs from organisms in the aquatic environment [20,58,59,60]. Differentiated shifts in fatty acid profile were also observed between the tested species in this study due to exudate exposure. Thes obtained results demonstrated 13 and 10 differentiating fatty acids in *G. umbilicalis’* whole body and *P. elegans’* hepatopancreas, respectively, indicating *A. armata* exudate altered fatty acid biosynthesis and metabolism in these organisms, although through a different way.

When looking to the seventeen significantly responsive FAs to the exudate, polyunsaturated fatty acids (PUFA) tended to increase in both species after exposure, while for saturated and monounsaturated FAs, the opposite was observed. The increase in PUFA content can be considered a defense mechanism, protecting the membranes from oxidation disruption [58,61]. For *G. umbilicalis*, an increase in the first cluster including ARA (20:4 n6) and DPA (22:5 n3) can be seen. It is known that DPA can be retroconverted to eicosapentaenoic acid (EPA; 20:5 n3) and that it reacts with lipoxygenases to form distinctive oxylipins, such as the specialized pro-resolving mediators involved in the resolution of inflammation [62]. Additionally, n3 PUFAs like DPA serve as precursors of eicosanoids (prostaglandins, thromboxanes, leukotrienes, etc.), which have a wide range of physiological functions in immune system, inflammatory response, neural function, reproduction, and improve the organisms’ adaptation to environmental stress [63]. The increase of ARA (20:4 n6) in both invertebrates is an indication that this FA was possibly required for activation of eicosanoid synthesis for the regulation of inflammation and immunity responses [19,64]. Moreover, DGLA (20:3 n6) and EDA (20:2 n6), although clustered in a different group, presented an increase, particularly at 0.47% (C5). These particular FAs are also involved in eicosanoid synthesis, and DGLA is also desaturated to form ARA, thus explaining their increase.

For both organisms, the sat./insat. ratio decreased significantly for the higher concentrations of *A. armata* exudate tested, when the clustered FAs were considered. An increase in n3 FAs was observed for *G. umbilicalis* and in n6 for *P. elegans*, while a decrease in SFAs was observed for both species (Tables S3 and S4). This may be attributed to an inflammatory response due to the increase of n3 and n6 FAs, as previously reported by Simopoulos [65]. This type of PUFAs serve as potent anti-inflammatory (n3), pro-inflammatory (n6), and immunomodulatory agents. HPA (21:5 n3) is a stronger inhibitor of the conversion of α-linoleic acid and dihomo-γ-linolenic acid to ARA (20:4 n6) and inactivates prostaglandin H synthase as rapidly as do ARA [66].

Considering the clustered FAs, although there is a trend to an increase in PUFA with increasing exudate concentrations in *P. elegans*, at the higher concentration (2.46%, C6) there is a slight trend to the decrease in n3 PUFA. This trend may be due to the loss of ability of *P. elegans* to cope with the stressor at higher concentrations, leading to the observed increase in lipid peroxidation levels. However, and most likely, the pro-oxidant effect of exudate altered membrane integrity and fluidity in the shrimp membrane cells, due to decreased adaptation and activity of the membrane-bound enzymes and pumps that block the membrane permeability. At the same concentration, despite the overall PUFA reduction, n6 and short chain FAs increased. In the literature, these specific FAs are correlated to a pro-inflammatory response, by inducing inflammatory lipid mediators and the production of cytokines [19,64,67], overlapping their concentrations to those FAs with anti-inflammatory effect (n3 PUFA). These results are in accordance, since more severe effects caused by higher stressor concentrations were expected, favoring the cellular inflammatory process.

Again, as discussed above, although there are common FA responses between species, the overall more evident effects seen in the shrimp might be due not only to the different range of toxic concentrations but also the tissues used—which was the same reasoning as for the biomarkers.

## 5. Conclusions

In the present study, potential neurotoxicity, oxidative stress mechanisms of action and fatty acid profile changes behind the previously reported impacts of *A. armata* exudate on these two rock-pool invertebrate species, i.e., mortality, behavior, and others [8] were addressed. In *P. elegans*, oxidative stress and neurotoxic effects seem to drive to toxicity and effects in higher levels of biological organization (e.g., mortality) [8], while for *G. umbilicalis* these routes do not seem to relate to seen impacts (e.g., survival and feeding behavior) [8]. Fatty acids also revealed different metabolic responses in the different tissues of the different species, despite the increase of ARA and DPA in both invertebrates, which points toward common inflammatory and immunity regulation responses.

These effects induced by *Asparagopsis armata* on marine invertebrates’ points toward the threat that this invasive seaweed represents in organisms. Exudate toxicity is maximized in sites where water is poorly mixed, allowing released algal products to concentrate in tide pools and where these organisms are adjacent to the releasor (e.g., surfaces of thalli), which will have implications in the distribution and abundance of these species with important ecological roles.

## Supplementary Materials

The following are available online at https://www.mdpi.com/2079-7737/10/3/223/s1, Table S1: Fatty acid composition found in *Gibbula umbilicalis* exposed to *Asparagopsis armata* exudate for 168 h, plus control., Table S2: Fatty acid composition found in *Palaemon elegans* exposed to *Asparagopsis armata* exudate for 168 h, plus control., Table S3: Differences between FA classes across the clustered FAs found in *Gibbula umbilicalis* exposed to *Asparagopsis armata* exudate for 168 h, plus control., Table S4: Differences between FA classes across the clustered FAs found in *Palaemon elegans* exposed to *Asparagopsis armata* exudate for 168 h, plus control.
