# Supplementary material for: Asparagopsis armata Exudate Cocktail: The Quest for the Mechanisms of Toxic Action of an Invasive Seaweed on Marine Invertebrates
Source: Biology (Basel). 2021 Mar 14;10(3):223. doi: 10.3390/biology10030223 (PMC8002046; doi:10.3390/biology10030223)
Supplement: Supplementary file 1 [file biology-10-00223-s001.pdf]

biology

MDPI

Supplementary Materials

Asparagopsis armata Exudate Cocktail: The Quest for the Mechanisms of Toxic Action of an Invasive Seaweed on Marine Invertebrates

Carla O. Silva, Tiago Simões, Rafael Félix, Amadeu M.V.M. Soares, Carlos Barata, Sara C. Novais, and Marco F.L. Lemos

Table S1. - Fatty acid composition found in *Gibbula umbilicalis* exposed to *Asparagopsis armata* exudate for 168h, plus control. Data are expressed as mg/g (mean ± standard error). Statistical differences to control (by the Nemenyi post-hoc test) are highlighted (* p<0.05; ** p<0.01; *** p<0.001). SFA = saturated fatty acid; MUFA = monounsaturated fatty acid; PUFA = polyunsaturated fatty acid.

		Asparagopsis armata Exudate (%)						
**FA**		**0**	**0.04**	**0.07**	**0.14**	**0.25**	**0.47**	**0.87**
SFA	C 10:0	0.09 ± 0.01	0.06 ± 0.01	0.06 ± 0.01	0.06 ± 0.01	0.04 ± 0.01	0.05 ± 0.01 **	0.03 ± 0.00 **
	C 12:0	0.52 ± 0.29	0.82 ± 0.28	0.94 ± 0.56	0.45 ± 0.23	0.68 ± 0.22	1.13 ± 0.35	0.31 ± 0.17
	C 13:0	0.08 ± 0.02	0.11 ± 0.03	0.30 ± 0.12	0.23 ± 0.07	0.22 ± 0.06	0.07 ± 0.02	0.07 ± 0.02
	C 14:0	0.76 ± 0.12	0.87 ± 0.16	0.99 ± 0.28	0.69 ± 0.07	0.70 ± 0.09	1.19 ± 0.13	0.56 ± 0.03
	C 15:0	0.07 ± 0.01	0.06 ± 0.01	0.09 ± 0.03	0.08 ± 0.00	0.07 ± 0.01	0.11 ± 0.02	0.06 ± 0.01
	C 16:0	7.15 ± 1.01	7.46 ± 1.12	8.16 ± 1.22	8.08 ± 1.14	7.36 ± 0.53	8.64 ± 1.47	7.39 ± 1.48
	C 17:0	0.04 ± 0.02	0.03 ± 0.01	0.02 ± 0.01	0.03 ± 0.01	0.03 ± 0.01	0.04 ± 0.02	0.04 ± 0.01
	C 18:0	5.98 ± 0.62	6.90 ± 1.48	6.98 ± 0.95	7.92 ± 1.64	6.67 ± 0.30	7.07 ± 1.18	6.26 ± 1.05
	C 20:0	0.00 ± 0.00	0.00 ± 0.00	0.00 ± 0.00	0.00 ± 0.00	0.00 ± 0.00	0.00 ± 0.00	0.00 ± 0.00
	C 22:0	0.02 ± 0.01	0.08 ± 0.06	0.01 ± 0.01	0.01 ± 0.00	0.00 ± 0.00*	0.01 ± 0.00	0.00 ± 0.00*
	C 24:0	0.11 ± 0.02	0.10 ± 0.03	0.10 ± 0.05	0.07 ± 0.02	0.14 ± 0.06	0.08 ± 0.03	0.04 ± 0.01
∑SFA		14.69 ± 1.64	16.38 ± 2.53	17.67 ± 2.87	17.54 ± 2.61	15.77 ± 0.55	18.30 ± 2.51	14.72 ± 2.41
MUFA	C 14:1	0.05 ± 0.01	0.02 ± 0.01	0.02 ± 0.00	0.01 ± 0.00	0.00 ± 0.00*	0.02 ± 0.01	0.01 ± 0.00
	C 15:1	0.01 ± 0.00	0.00 ± 0.00	0.01 ± 0.00	0.01 ± 0.01	0.01 ± 0.00	0.02 ± 0.01	0.01 ± 0.00
	C 16:1 n5	0.03 ± 0.01	0.04 ± 0.01	0.04 ± 0.01	0.08 ± 0.01	0.07 ± 0.01	0.14 ± 0.04 ***	0.10 ± 0.02 ***
	C 16:1 n7	0.25 ± 0.13	0.14 ± 0.03	0.13 ± 0.03	0.22 ± 0.08	0.15 ± 0.02	0.32 ± 0.12	0.12 ± 0.02
	C 16:1 n9	0.01 ± 0.00	0.01 ± 0.00	0.01 ± 0.00	0.01 ± 0.00	0.01 ± 0.00	0.04 ± 0.03	0.01 ± 0.00
	C 17:1	0.02 ± 0.01	0.01 ± 0.00	0.01 ± 0.00	0.01 ± 0.01	0.01 ± 0.00	0.01 ± 0.00	0.02 ± 0.01
	C 18:1 n7	0.01 ± 0.00	0.05 ± 0.02*	0.03 ± 0.02	0.03 ± 0.01	0.02 ± 0.01	0.08 ± 0.04	0.03 ± 0.01
	C 18:1 n9	1.95 ± 0.37	2.47 ± 0.73	2.97 ± 1.27	1.53 ± 0.38	3.19 ± 1.23	1.69 ± 0.19	1.22 ± 0.36
	C 20:1 n9	0.27 ± 0.13	0.17 ± 0.03	0.21 ± 0.02	0.13 ± 0.05	0.15 ± 0.03	0.13 ± 0.02*	0.15 ± 0.04
	C 22:1 n9	0.01 ± 0.00	0.01 ± 0.00	0.05 ± 0.04	0.02 ± 0.02	0.00 ± 0.00	0.06 ± 0.05	0.02 ± 0.01
	C 24:1 n9	0.18 ± 0.07	0.13 ± 0.06	0.13 ± 0.06	0.02 ± 0.01	0.01 ± 0.00	0.11 ± 0.06	0.13 ± 0.05
∑MUFA		2.79 ± 0.45	3.04 ± 0.79	3.60 ± 1.35	2.08 ± 0.40	3.62 ± 1.24	2.63 ± 0.35	1.80 ± 0.42
PUFA	C 16:2 n4	0.10 ± 0.02	0.12 ± 0.03	0.12 ± 0.02	0.14 ± 0.03	0.10 ± 0.02	0.13 ± 0.03	0.16 ± 0.05
	C 16:2 n7	0.00 ± 0.00	0.00 ± 0.00	0.00 ± 0.00	0.00 ± 0.00	0.00 ± 0.00	0.00 ± 0.00	0.00 ± 0.00
	C 18:2 n6	0.69 ± 0.14	0.76 ± 0.12	0.92 ± 0.20	0.56 ± 0.07	0.69 ± 0.22	0.87 ± 0.19	0.64 ± 0.09
	C 16:3 n4	0.09 ± 0.08	0.00 ± 0.00	0.00 ± 0.00	0.00 ± 0.00	0.00 ± 0.00	0.00 ± 0.00	0.01 ± 0.00
	C 18:3 n6	0.01 ± 0.00	0.03 ± 0.01	0.04 ± 0.03	0.010.00	0.03 ± 0.01	0.08 ± 0.05	0.02 ± 0.01
	C 18:3 n3	0.05 ± 0.01	0.15 ± 0.06	0.10 ± 0.04	0.10 ± 0.04	0.07 ± 0.02	0.17 ± 0.04	0.06 ± 0.02
	C 18:4 n1	0.02 ± 0.00	0.01 ± 0.00	0.04 ± 0.02	0.02 ± 0.00	0.03 ± 0.02	0.02 ± 0.01	0.01 ± 0.00
	C 18:4 n3	0.04 ± 0.03	0.30 ± 0.18	0.09 ± 0.01	0.26 ± 0.16	0.19 ± 0.06	0.14 ± 0.03	0.10 ± 0.02
	C 20:2 n6	0.26 ± 0.10	0.27 ± 0.18	0.48 ± 0.27	0.06 ± 0.02 *	0.12 ± 0.05	0.34 ± 0.08	0.21 ± 0.06
	C 20:3 n3	0.02 ± 0.01	0.01 ± 0.00	0.01 ± 0.00	0.01 ± 0.00	0.01 ± 0.00	0.01 ± 0.00	0.01 ± 0.00

C 20:3 n6	0.12 ± 0.01	0.13 ± 0.02	0.13 ± 0.02	0.08 ± 0.01	0.07 ± 0.01 *	0.16 ± 0.03	0.10 ± 0.01
C 20:4 n3	0.09 ± 0.07	0.07 ± 0.05	0.05 ± 0.02	0.09 ± 0.08	0.21 ± 0.08	0.04 ± 0.02	0.07 ± 0.05
C 20:4 n6	0.20 ± 0.04	0.20 ± 0.02	0.24 ± 0.06	0.47 ± 0.06	0.46 ± 0.03	0.77 ± 0.14 ***	0.55 ± 0.09 ***
C 20:5 n3	0.38 ± 0.10	0.53 ± 0.10	0.38 ± 0.08	0.64 ± 0.05	0.57 ± 0.06	1.00 ± 0.28	0.65 ± 0.24
C 21:5 n3	0.00 ± 0.00	0.01 ± 0.00	0.00 ± 0.00	0.00 ± 0.00*	0.01 ± 0.00	0.01 ± 0.00	0.00 ± 0.00
C 22:2 n6	0.02 ± 0.01	0.01 ± 0.00	0.02 ± 0.01	0.01 ± 0.01	0.01 ± 0.00	0.04 ± 0.02	0.02 ± 0.01
C 22:3 n6	0.02 ± 0.01	0.02 ± 0.01	0.00 ± 0.00 *	0.03 ± 0.01	0.00 ± 0.00	0.00 ± 0.00	0.01 ± 0.00
22:4 n6	0.01 ± 0.00	0.00 ± 0.00	0.02 ± 0.01	0.03 ± 0.01	0.05 ± 0.01 **	0.05 ± 0.04	0.03 ± 0.01
C 22:5 n3	0.08 ± 0.02	0.13 ± 0.02	0.13 ± 0.03	0.30 ± 0.04 **	0.29 ± 0.01 **	0.45 ± 0.13 **	0.22 ± 0.03
22:5 n6	0.01 ± 0.00	0.03 ± 0.01	0.02 ± 0.01	0.01 ± 0.00	0.02 ± 0.01	0.03 ± 0.02	0.02 ± 0.01
C 22:6 n3	0.22 ± 0.07	0.48 ± 0.14	0.25 ± 0.11	0.40 ± 0.05	0.36 ± 0.11	0.64 ± 0.23	0.74 ± 0.38
∑PUFA	2.56 ± 0.45	3.25 ± 0.38	3.01 ± 0.67	1.43 ± 0.18	3.26 ± 0.36	4.91 ± 0.93	3.62 ± 0.88
Unsat./Sat	0.36 ± 0.02	0.42 ± 0.05	0.35 ± 0.04	0.33 ± 0.04	0.44 ± 0.07	0.41 ± 0.03	0.39 ± 0.06
n3	1.04 ± 0.30	1.68 ± 0.32	1.01 ± 0.20	1.80 ± 0.25	1.70 ± 0.25	2.45 ± 0.64	1.86 ± 0.64
n6	1.34 ± 0.27	1.45 ± 0.30	1.88 ± 0.55	1.26 ± 0.07	1.46 ± 0.25	2.33 ± 0.32	1.59 ± 0.23
n7	0.26 ± 0.13	14.40 ± 2.55 ***	0.16 ± 0.05	0.25 ± 0.08	0.17 ± 0.02	0.40 ± 0.15	0.15 ± 0.03
n9	2.41 ± 0.40	0.58 ± 0.14 **	3.37 ± 1.30	1.71 ± 0.39	3.35 ± 1.23	2.03 ± 0.26	1.53 ± 0.39
n3/n6	0.85 ± 0.20	1.48 ± 0.37	0.68 ± 0.18	1.46 ± 0.24	1.32 ± 0.26	1.00 ± 0.20	1.07 ± 0.22
N	7	6	6	7	7	6	6

Table S2 - Fatty acid composition found in *Palaemon elegans* exposed to *Asparagopsis armata* exudate for 168h, plus control. Data are expressed as mg/g (mean ± standard error). Statistical differences to control (by the Nemenyi post-hoc test) are highlighted (* $p < 0.05$; ** $p < 0.01$; *** $p < 0.001$). SFA = saturated fatty acid; MUFA = monounsaturated fatty acid; PUFA = polyunsaturated fatty acid.

		***Asparagopsis armata* Exudate (%)**						
**FA**		**0**	**0.11**	**0.21**	**0.39**	**0.72**	**1.33**	**2.46**
SFA	C 10:0	0.06 ± 0.02	0.05 ± 0.02	0.04 ± 0.01	0.04 ± 0.01	0.11 ± 0.02 **	0.06 ± 0.01	0.07 ± 0.01
	C 12:0	1.12 ± 0.57	0.33 ± 0.16	0.30 ± 0.11	1.09 ± 0.42	0.62 ± 0.20	0.35 ± 0.21	0.57 ± 2.69
	C 13:0	0.59 ± 0.22	0.27 ± 0.07	0.08 ± 0.02	0.08 ± 0.03	0.07 ± 0.02 ***	0.07 ± 0.01 ***	0.04 ± 0.01 ***
	C 14:0	0.83 ± 0.27	0.43 ± 0.08	0.24 ± 0.05	0.79 ± 0.27	0.60 ± 0.12	0.42 ± 0.14	0.57 ± 0.13
	C 15:0	0.05 ± 0.01	0.04 ± 0.01	0.03 ± 0.00	0.05 ± 0.01	0.06 ± 0.01	0.04 ± 0.01	0.08 ± 0.03
	C 16:0	7.15 ± 1.44	5.21 ± 0.46	3.68 ± 0.44	5.48 ± 1.26	5.49 ± 1.13	4.49 ± 0.65	5.67 ± 0.95
	C 17:0	0.03 ± 0.00	0.02 ± 0.00	0.01 ± 0.00	0.02 ± 0.01	0.03 ± 0.01	0.02 ± 0.00	0.03 ± 0.01
	C 18:0	7.89 ± 1.95	5.32 ± 0.66	3.96 ± 0.64	7.25 ± 1.81	5.34 ± 0.91	4.65 ± 0.78	5.64 ± 0.90
	C 20:0	0.00 ± 0.00	0.00 ± 0.00	0.00 ± 0.00	0.00 ± 0.00	0.00 ± 0.00	0.00 ± 0.00	0.02 ± 0.02
	C 22:0	0.10 ± 0.05	0.01 ± 0.00	0.01 ± 0.00	0.03 ± 0.01	0.03 ± 0.01	0.02 ± 0.01	0.15 ± 0.12
	C 24:0	0.01 ± 0.00	0.07 ± 0.03	0.04 ± 0.02	0.01 ± 0.00	0.01 ± 0.00	0.01 ± 0.01	0.13 ± 0.08
∑SFA		11.26 ± 2.48	11.69 ± 1.23	8.35 ± 1.02	14.82 ± 3.59	12.35 ± 2.13	10.11 ± 1.70	11.26 ± 2.48
MUFA	C 14:1	0.01 ± 0.01	0.00 ± 0.00	0.01 ± 0.01	0.01 ± 0.00	0.02 ± 0.00	0.01 ± 0.00	0.01 ± 0.01
	C 15:1	0.00 ± 0.00	0.00 ± 0.00	0.00 ± 0.00	0.00 ± 0.00	0.01 ± 0.00	0.00 ± 0.00	0.04 ± 0.01 **
	C 16:1 n5	0.03 ± 0.01	0.03 ± 0.00	0.02 ± 0.00	0.02 ± 0.01	0.03 ± 0.01	0.02 ± 0.00	0.03 ± 0.01
	C 16:1 n7	0.23 ± 0.07	0.13 ± 0.06	0.07 ± 0.01	0.09 ± 0.02	0.16 ± 0.04	0.11 ± 0.03	0.14 ± 0.02
	C 16:1 n9	0.03 ± 0.02	0.01 ± 0.00	0.00 ± 0.00	0.01 ± 0.00	0.01 ± 0.00	0.00 ± 0.00	0.01 ± 0.00
	C 17:1	0.01 ± 0.01	0.01 ± 0.00	0.01 ± 0.00	0.01 ± 0.00	0.02 ± 0.00	0.01 ± 0.00	0.02 ± 0.00
	C 18:1 n7	0.05 ± 0.02	0.03 ± 0.01	0.01 ± 0.01	0.01 ± 0.00	0.04 ± 0.01	0,02 ± 0.00	0.04 ± 0.01
	C 18:1 n9	2.84 ± 1.45	1.48 ± 0.21	0.51 ± 0.17	1.37 ± 0.41	1.77 ± 0.49	1.16 ± 0.24	0.01 ± 0.00
	C 20:1 n9	0.02 ± 0.01	0.02 ± 0.01	0.00 ± 0.00	0.01 ± 0.00	0.00 ± 0.00	0.00 ± 0.00 **	0.00 ± 0.00 **
	C 22:1 n9	0.01 ± 0.00	0.01 ± 0.01	0.00 ± 0.00	0.05 ± 0.04	0.01 ± 0.00	0.00 ± 0.00	0.00 ± 0.00
	C 24:1 n9	0.09 ± 0.03	0.16 ± 0.05	0.09 ± 0.03	0.11 ± 0.03	0.18 ± 0.11	0.08 ± 0.03	0.08 ± 0.04
∑MUFA		3.31 ± 1.54	1.88 ± 0.26	0.73 ± 0.18	1.70 ± 0.44	2.25 ± 0.59	1.42 ± 0.26	2.64 ± 0.74
PUFA	C 16:2 n4	0.15 ± 0.04	0.11 ± 0.01	0.08 ± 0.01	0.13 ± 0.04	0.14 ± 0.03	0,10 ± 0.02	0.13 ± 0.02
	C 16:2 n7	0.00 ± 0.00	0.00 ± 0.00	0.00 ± 0.00	0.00 ± 0.00	0.00 ± 0.00	0,00 ± 0.00	0.00 ± 0.00
	C 18:2 n6	0.88 ± 0.27	0.48 ± 0.05	0.22 ± 0.03	0.67 ± 0.30	1.30 ± 0.64	0.38 ± 0.08	0.43 ± 0.10
	C 16:3 n4	0.03 ± 0.01	0.02 ± 0.00	0.02 ± 0.00	0.03 ± 0.01	0.02 ± 0.00	0.01 ± 0.00	0.02 ± 0.00

C 18:3 n6	0.02 ± 0.01	0.01 ± 0.01	0.01 ± 0.01	0.01 ± 0.01	0.02 ± 0.01	0.03 ± 0.01	0.11 ± 0.08
C 18:3 n3	0.07 ± 0.02	0.05 ± 0.01	0.01 ± 0.00*	0.03 ± 0.02	0.09 ± 0.03	0.05 ± 0.02	0.03 ± 0.01
C 18:4 n1	0.15 ± 0.04	0.12 ± 0.13	0.08 ± 0.01	0.10 ± 0.02	0.20 ± 0.04	0.12 ± 0.01	0.16 ± 0.02
C 18:4 n3	0.26 ± 0.18	0.17 ± 0.08	0.10 ± 0.04	0.17 ± 0.10	0.09 ± 0.03	0.12 ± 0.05	0.19 ± 0.06
C 20:2 n6	0.40 ± 0.29	0.08 ± 0.02	0.08 ± 0.05	0.20 ± 0.10	0.20 ± 0.06	0.07 ± 0.04	0.08 ± 0.04
C 20:3 n3	0.02 ± 0.01	0.01 ± 0.01	0.01 ± 0.00	0.01 ± 0.00	0.02 ± 0.00	0.01 ± 0.00	0.03 ± 0.02
C 20:3 n6	0.07 ± 0.07	0.07 ± 0.03	0.05 ± 0.01	0.08 ± 0.02	0.07 ± 0.01	0.05 ± 0.01	0.07 ± 0.01
C 20:4 n3	0.02 ± 0.01	0.13 ± 0.27	0.05 ± 0.02	0.02 ± 0.02	0.02 ± 0.01	0.07 ± 0.05	0.15 ± 0.07
C 20:4 n6	0.12 ± 0.05	0.14 ± 0.07	0.06 ± 0.02	0.05 ± 0.02	0.21 ± 0.03 *	0.10 ± 0.02	0.14 ± 0.03
C 20:5 n3	0.68 ± 0.23	0.59 ± 0.34	0.32 ± 0.09	0.30 ± 0.08	0.83 ± 0.17	0.40 ± 0.09	0.58 ± 0.15
C 21:5 n3	0.01 ± 0.00	0.00 ± 0.00	0.01 ± 0.00	0.02 ± 0.01	0.01 ± 0.00 **	0.00 ± 0.00	0.00 ± 0.00
C 22:2 n6	0.03 ± 0.01	0.01 ± 0.01	0.02 ± 0.01	0.01 ± 0.00	0.09 ± 0.03 **	0.07 ± 0.02	0.06 ± 0.02
C 22:3 n6	0.00 ± 0.00	0.00 ± 0.00	0.00 ± 0.00	0.00 ± 0.00	0.01 ± 0.00 *	0.00 ± 0.00	0.01 ± 0.00 *
22:4 n6	0.04 ± 0.04	0.00 ± 0.00	0.00 ± 0.00	0.00 ± 0.00	0.00 ± 0.00	0.00 ± 0.00	0.00 ± 0.00
C 22:5 n3	0.03 ± 0.00	0.05 ± 0.04	0.02 ± 0.01	0.04 ± 0.02	0,13 ± 0.03 **	0.06 ± 0.01	0.03 ± 0.01
22:5 n6	0.01 ± 0.01	0.00 ± 0.00	0.00 ± 0.00	0.00 ± 0.00	0.03 ± 0.01	0.00 ± 0.00	0.01 ± 0.00
C 22:6 n3	0.28 ± 0.06	0.29 ± 0.15	0.15 ± 0.05	0.14 ± 0.06	0.55 ± 0.16	0.29 ± 0.09	0.38 ± 0.10
∑PUFA	3.21 ± 0.79	2.23 ± 0.32	1.21 ± 0.14 **	1.94 ± 0.51	3.85 ± 0.80	1.83 ± 0.37	3.60 ± 0.96
Unsat./Sat	0.36 ± 0.07	0.35 ± 0.02	0.26 ± 0.05	0.26 ± 0.04 *	0.47 ± 0.06	0.32 ± 0.02	1.25 ± 0.86
n3	1.38 ± 0.36	1.29 ± 0.27	0.67 ± 0.14 *	0.75 ± 0.17	1.75 ± 0.33	1.02 ± 0.28	2.50 ± 0.93
n6	1.65 ± 0.63	0.80 ± 0.08	0.45 ± 0.08	1.04 ± 0.37	1.95 ± 0.69	0.70 ± 0.15	0.94 ± 0.13
n7	0.28 ± 0.08	0.15 ± 0.03	0.09 ± 0.02	0.10 ± 0.03	0.21 ± 0.04	0.13 ± 0.03	0.18 ± 0.03
n9	2.98 ± 1.49	1.68 ± 0.25	0.61 ± 0.17	0.27 ± 0.08 ***	0.79 ± 0.18	0.44 ± 0.10	0.73 ± 0.10
n3/n6	1.32 ± 0.56	1.61 ± 0.28	1.84 ± 0.43	1.75 ± 0.72	1.52 ± 0.35	1.99 ± 0.50	2.81 ± 1.03
N	5	8	7	7	7	8	7

Table S3. Differences between FA classes across the clustered FAs found in *Gibbula umbilicalis* exposed to *Asparagopsis armata* exudate for 168h, plus control. Data are expressed as mg/g (mean ± standard error). Statistical differences to control (by the Nemenyi post-hoc test) are highlighted (* $p < 0.05$; ** $p < 0.01$; *** $p < 0.001$). SFA = saturated fatty acid; MUFA = monounsaturated fatty acid; PUFA = polyunsaturated fatty acid.

**FA**	***Asparagopsis armata* exudate (%)**						
	**0**	**0.04**	**0.07**	**0.14**	**0.25**	**0.47**	**0.87**
∑SFA	0.16 ± 0.06	0.15 ± 0.2	0.09 ± 0.04	0.08 ± 0.03	0.05 ± 0.02 **	0.08 ± 0.03	0.04 ± 0.00 8**
∑MUFA	0.36 ± 0.09	0.28 ± 0.11	0.3 ± 0.07	0.26 ± 0.08	0.24 ± 0.10	0.37 ± 0.26	0.29 ± 0.13
∑PUFA	0.69 ± 0.38	0.67 ± 0.58	1.02 ± 0.88	0.98 ± 0.26	1.0 ± 0.19	1.78 ± 0.72 *	1.1 ± 0.43
Sat/Insat.	0.16 ± 0.05	0.18 ± 0.24	0.09 ± 0.06	0.07 ± 0.03	0.04 ± 0.02 **	0.04 ± 0.01 **	0.04 ± 0.02 **
n3	0.08 ± 0.04	0.14 ± 0.07	0.13 ± 0.07	0.3 ± 0.09 **	0.29 ± 0.03 **	0.45 ± 0.32 *	0.22 ± 0.08
n6	0.6 ± 0.36	0.62 ± 0.57	0.88 ± 0.82	0.67 ± 0.20	0.71 ± 0.18	1.32 ± 0.45	0.89 ± 0.37
n3/n6	0.16 ± 0.09	0.31 ± 0.21	0.19 ± 0.09	0.46 ± 0.11 **	0.43 ± 0.11 *	0.32 ± 0.18	0.25 ± 0.05
N	7	6	6	7	7	6	6

Table S4. Differences between FA classes across the clustered FAs found in *Palaemon elegans* exposed to *Asparagopsis armata* exudate for 168h, plus control. Data are expressed as mg/g (mean ± standard error). Statistical differences to control (by the Nemenyi post-hoc test) are highlighted (* $p < 0.05$; ** $p < 0.01$; *** $p < 0.001$). SFA = saturated fatty acid; MUFA = mono-unsaturated fatty acid; PUFA = polyunsaturated fatty acid.

**FA**	***Asparagopsis armata* Exudate (%)**						
	**0**	**0.11**	**0.21**	**0.39**	**0.72**	**1.33**	**2.46**
∑SFA	0.66 ± 0.48	0.32 ± 0.21	0.12 ± 0.07 *	0.12 ± 0.09 *	0.2 ± 0.09	0.14 ± 0.06 *	0.11 ± 0.06 *
∑MUFA	0.02 ± 0.02	0.02 ± 0.02	0.006 ± 0.008	0.014 ± 0.013	0.009 ± 0.008	0.0 ± 0.0	0.04 ± 0.04
∑PUFA	0.14 ± 0.11	0.20 ± 0.08	0.1 ± 0.06	0.12 ± 0.08	0.47 ± 0.11	0.25 ± 0.13	0.26 ± 0.09
Sat/Insat.	3.15 ± 2.25	1.53 ± 1.03	1.35 ± 0.69	1.09 ± 0.59	0.4 ± 0.15 **	0.61 ± 0.22	0.38 ± 0.19 **
n3	0.11 ± 0.05	0.1 ± 0.03	0.04 ± 0.02	0.10 ± 0.10	0.26 ± 0.13	0.12 ± 0.08	0.07 ± 0.04
n6	0.16 ± 0.01	0.15 ± 0.08	0.08 ± 0.05	0.07 ± 0.04	0.36 ± 0.11 *	0.18 ± 0.09	0.32 ± 0.07*
n3/n6	2.21 ± 3.41	0.85 ± 0.52	0.70 ± 0.47	1.46 ± 1.36	0.82 ± 0.53	0.63 ± 0.25	0.31 ± 0.16
N	5	8	7	7	7	8	7
